# In Vitro and In Vivo Applications of Magnesium-Enriched Biomaterials for Vascularized Osteogenesis in Bone Tissue Engineering: A Review of Literature

**DOI:** 10.3390/jfb14060326

**Published:** 2023-06-19

**Authors:** Jie Hu, Jiahui Shao, Gan Huang, Jieyuan Zhang, Shuting Pan

**Affiliations:** Department of Oral and Maxillofacial Surgery, The First Affiliated Hospital of Nanchang University, Nanchang 330006, China; 403006210068@email.ncu.edu.cn (J.H.); 15717927958@163.com (J.S.); hg3946@163.com (G.H.);

**Keywords:** magnesium, bone tissue engineering, vascularized osteogenesis, bone defects

## Abstract

Bone is a highly vascularized tissue, and the ability of magnesium (Mg) to promote osteogenesis and angiogenesis has been widely studied. The aim of bone tissue engineering is to repair bone tissue defects and restore its normal function. Various Mg-enriched materials that can promote angiogenesis and osteogenesis have been made. Here, we introduce several types of orthopedic clinical uses of Mg; recent advances in the study of metal materials releasing Mg ions (pure Mg, Mg alloy, coated Mg, Mg-rich composite, ceramic, and hydrogel) are reviewed. Most studies suggest that Mg can enhance vascularized osteogenesis in bone defect areas. Additionally, we summarized some research on the mechanisms related to vascularized osteogenesis. In addition, the experimental strategies for the research of Mg-enriched materials in the future are put forward, in which clarifying the specific mechanism of promoting angiogenesis is the crux.

## 1. Introduction

As a pivotal structure in vertebrates, bone can be easily aggrieved by accidents, diseases, and aging [1,2,3,4]. However, the self-healing ability of bone is limited. For microfractures, bone has sufficient self-healing ability. Unfortunately, for fractures exceeding the critical size, bone cannot form complete fracture healing [2]. Presently, the gold standard for the treatment of critical bone defects is still autologous bone transplantation, which brings secondary injury such as donor site deformity, hypersensitivity, and muscle weakness, and bleeding, chronic pain, inflammation, and infection may also occur after operation. Therefore, finding suitable bone substitutes is still the critical issue of bone tissue engineering (BTE) [2,5,6]. The regeneration of large bone defects requires rapid angiogenesis to provide nutrients and oxygen, and inadequate angiogenesis often leads to undesirable bone regeneration, which is determined by the complex structure of long bones containing a peripheral cortical shell with a network of vascular penetration channels and an internal highly vascularized bone marrow space [7]. Appropriate blood supply is the key element to ensure fracture healing [8,9]; the lack of a functional microvasculature connected to the host blood supply is responsible for implant failure [9,10]. The global rate of fracture nonunion is about 10% [7]. Notably, when fracture is accompanied by macrovascular injury, the rate of fracture nonunion rises rapidly to 46% [11].

Ever since magnesium (Mg) was isolated in 1808, it has been widely used in many fields [12,13,14,15,16,17]. As the fourth most abundant cation in the human body, Mg plays a crucial role in metabolism [18]. It was first used in biomaterials until the mid-nineteenth century [17]. Recently, functional Mg materials have shown great advantages over traditional materials in many fields, including bio-Mg materials [19]. Recent studies have shown that Mg has the potential to promote osseointegration and angiogenesis [20]. Thus, the applications of modern Mg-based biomaterials can be mainly divided into two areas: vascular application and orthopedic application [17]. Mg and Mg-based alloys are potential materials for bone repair and coronary arterial stents due to their excellent biocompatibility and biodegradability [21,22,23,24,25,26]. Mg-based alloys have been used in implantable vascular stents and Mg-based cardiovascular stents with encouraging clinical trial outcomes [19,27]. The degradation rate of physiological environment once limited the application of Mg-based alloys in bone repair. However, with the development of biomaterials, such as alloying and surface coating strategy, which can delay the degradation of Mg, Mg and Mg-based alloys are gradually becoming candidates for a new generation of orthopedic implants.

At present, there are three main defects in a variety of metal biological implant materials. Firstly, the corrosion or wear products have biological toxicity, causing inflammatory cascades of the organism. Secondly, the stress shielding effect may result in the absorption of surrounding healthy bone, thus decreasing the stability of the implant. Thirdly, some materials need to be removed with a secondary surgery [18].

Bone is an artistic craft of nature. It is a strong composite material composed of weak ingredients, with a seven-order hierarchical structure [28]. Beyond all doubt, autologous bone is more suitable for the human body than any artificial material in any aspect. Consequently, reproducing and mimicking the microenvironment in vivo is essential for the osteogenesis of stem cells [29]. The fabrication of metal scaffold materials has been committed to imitating the osteogenic environment in the human body. Mg is an active ingredient present in natural bone matrix [30]; therefore, Mg has the potential to become a bioactive ingredient in simulating the osteogenic microenvironment. Mg transporters are highly expressed in the endochondral ossification region of the cartilage in mouse embryos, endochondral ossification is a vascularized bone formation process, suggesting that Mg may be closely related to vascularized osteogenesis [30].

## 2. Mg-Based Orthopedic Implants for Clinical Use

The advantages of Mg-based materials in bone implants over the currently used permanent metal implants are: biodegradability, biocompatibility, mechanical strength close to cortical bone, and osteoinductivity [4]. The earliest use of Mg-based orthopaedic materials for the successful treatment of human bone fracture was reported by Lambotte, who used Mg nails to fix supracondylar fractures in four children, and all the joint functions were fully restored without any complications [4]. Previously, Chlumský used high-purity Mg to prevent joint stiffness and restore joint motion in humans after bony separation of ankylotic joints [31]. Since then, other researchers have gradually used Mg-based materials for fracture treatment, but the attention received by Mg-based materials has slowly waned due to uncontrollable Mg corrosion in vivo [31]. With the rapid development of the metallurgy field, scientists began to focus again on the value of Mg as a biodegradable metal for clinical application in recent years [31]. At present, Mg-based materials are divided into two types in clinical applications: cardiovascular and orthopedic biodegradable metals. This review focuses on the reported biodegradable metals for clinical use in orthopaedics.

There are two commercialized Mg-based orthopaedic materials which have been used in the clinical setting: MgYReZr (MAGNEZIX^®^ CS fabricated by Syntellix AG, Hanover, Germany) and Mg-Ca-Zn (K-MET screws developed by U&I Company, Uijeongbu-si, Republic of Korea) [3,32]. The high-purity Mg screw designed by Chinese researchers Zhao et al. and fabricated by Eontec in Dongguan, Guangdong has been allowed for clinical trial in 2019 by the Chinese National Medical Products Administration (NMPA), but has not been approved for clinical use yet [3,33]. The results of their previous randomized prospective clinical trials showed that pure Mg screws possess great potential in clinical medical applications [4,34]. Table 1 compares the mechanical properties of Mg-based materials for clinical use and bone.

### 2.1. MgYReZr (MAGNEZIX^®^ CS)

The first prospective, randomized, controlled clinical pilot study of MgYReZr was reported in Germany, which led to the MgYReZr compression screw MAGNEZIX^®^ CS gaining the Communauté Européenne (CE) mark in 2013 [3,39]. In this study, MgYReZr was used for fixation during chevron osteotomy in patients with a mild hallux valgus, indicating that MgYReZr is equivalent to a titanium screw in short-term clinical performance [39]. Furthermore, they also reported some in vivo and in vitro experimental results of the safety of MgYReZr: the upper part of the screw (diameter: 3 mm; length: 6 mm) was inserted into the left femoral supracondylar region of rabbits and observed for up to 12 months. The results showed that the screw had good biocompatibility and osteoconductivity, without acute, subacute, or chronic toxicity [40]. In another experiment, they implanted the MgYReZr pins into the femoral intercondylar notch of the stifle joint of rabbits for a maximum observation period of 12 weeks, and used the extracts of MgYReZr for cytotoxicity experiments. Experimental results suggest that the MgYReZr alloy has great potential as intra-articular degradable implants [41]. Subsequently, their further experiments confirmed that MgYReZr is promising for anterior cruciate ligament reconstruction, but the amount of gas liberated may need to be reduced [42,43]. As the first bioabsorbable metal implant for clinical use, MAGNEZIX^®^ CS was brought onto the market in 2015, setting a precedent [44]. After that, there have been more reports on other indications of MgYReZr, and its scope of application has also been extended to maxillofacial surgery [32,45,46,47,48,49,50]. Unfortunately, some patients who have been implanted with MgYReZr have reported extensive bone cysts and long bone healing time, demonstrating that MgYReZr may not be suitable for all fracture types [51].

### 2.2. Mg-Ca-Zn Screws

Korean researchers have designed Mg-Ca-Zn alloys and proven through experimentation that the new alloy has excellent biocompatibility, strength maintenance in vivo, mechanical properties, and an appropriate degradation rate, laying down a solid foundation for subsequent research [38,52,53]. By optimizing the degradation behavior and mechanical properties of Mg-Ca-Zn alloys, they finally concluded that the extruded Mg-5 wt%Ca-x wt%Zn (1 ≤ x ≤ 3) alloys had the desired performance as a biodegradable implant material [52]. After that, in April 2015, the Korean Food and Drug Administration(KFDA) approved Mg-Ca-Zn screws for clinical use [3]. Subsequently, they carried out a long-term (one-year follow-up) clinical study using Mg-5 wt%Ca-1 wt%Zn alloy screws. A total of 53 subjects were surgically fixed at the distal radius using this screw. After a one-year follow-up, all cases showed normal healing rates without any signs of pain [54].

## 3. Mg-Based Metal for Promoting Vascularized Osteogenesis

### 3.1. Pure Mg

Distraction osteogenesis (DO) is an effective operation for the treatment of large bone defects. It regenerates neo-formed bone and adjacent soft tissue by gradual and controlled traction of the bone segment separated by osteotomy [55]. The mechanical stretch produced in the process of DO causes a stronger angiogenic response than fracture healing [56]. In the early stage of DO, angiogenesis is also closely related to osteogenesis. Vascular endothelial cells play a core role in regulating bone regeneration of DO [57]. Ensuring blood supply is the key to successful osteogenesis in the process of DO [58]; this may be the key reason why the current research on pure Mg promoting vascularized osteogenesis focuses on DO.

Qin et al. [59] found that Mg could diffuse from the implant to the periosteum after the implantation of a 99.99%-pure Mg rod into the medullary cavity of a rat’s complete femur, and the amount of new bone formation in the femur increased significantly after implantation of Mg, but the pure Mg rod failed to fix the long-bone fracture of mice 3 weeks after implantation; this may be due to the weakening of mechanical strength over time caused by premature degradation. Alternatively, in another follow-up study [57], Mg nails with a purity of 99.99% were inserted into a femural midshaft bone defect (5 mm in length) model. Two weeks after stretching, they found that the generation of new bone increased by about 4-fold and the generation of new blood vessels increased by more than 5-fold. The in vivo studies show that Mg can up-regulate calcitonin gene-related peptide (CGRP)-mediated angiogenesis and thus promote osteogenesis. Similarly, in the rat DO model, Hamushan [60] et al. inserted a high-purity Mg pin (purity unknown) with a total length of 5 mm and diameter of 1 mm into the medullary cavity. Histological analysis confirmed that the Mg pin enhanced angiogenesis and bone consolidation in the experimental group. As such, pure Mg has a broad application prospect in DO.

### 3.2. Coated Mg

The low electrochemical standard potential of Mg determines its low corrosion resistance characteristics [61]. The rapid degradation of Mg and Mg alloys is an important reason for the nonunion of bone tissue after the implantation of Mg-based materials. Therefore, researchers have taken various methods to slow down the degradation rate of Mg, and the coating is one of the most effective methods [62]. The surface coating provides a corrosion barrier between the metal substrate and the corrosive medium, which can effectively improve corrosion resistance. In addition, the inherent characteristics of the metal substrate will not be negatively affected by the coating procedure [63], so the coated Mg has great development prospects. Cheng et al. [64] studied three films in common use, namely Mg hydroxide (Mg(OH)_2_), Mg fluoride (MgF_2_), and hydroxyapatite (HA) films. MgF_2_ showed the best ability of vascularized osteogenesis in vitro, but the results in vitro were different, which showed that HA film had optimal ability of bone integration.

Layered double hydroxides (LDHs) are promising bone implants, which can improve the corrosion resistance and biocompatibility of Mg alloys. Cheng et al. [65] prepared the Mg-Al LDH coating on the pure-Mg surface by hydrothermal treatment; Mg-Al LDH-coated Mg was more favorable for the osteogenic differentiation of mouse osteoblast cell line (MC3T3-E1) and promoted human umbilical vein endothelial cell (HUVEC) angiogenesis in vitro; in the in vivo femoral implantation experiments, Mg-Al LDH-coated Mg exhibited better osteointegration than bare Mg and Mg(OH)_2_-coated Mg. Significantly, this study also found that this new coated Mg-based material can induce macrophages to polarize anti-inflammatory M2 phenotype, and products of the induced macrophage can promote the osteogenic differentiation of rat bone marrow mesenchymal stem cells (rBMSC) and angiogenesis of HUVEC.

Microarc oxidation (MAO) is an electrochemical technique that can prepare anti-corrosion coating on the surface of Mg and Mg alloys, which can improve the corrosion resistance [63,66]. Previous studies have shown that the corrosion rate of MAO-treated Mg enhances biocompatibility [63]. Liang et al. [66] manufactured Cu^2+^-coated Mg-film by MAO, Cu-MAO Mg-enhanced osteogenesis and angiogenesis in a dose-dependent manner in vitro; they used a chicken embryo ex ovo model to be verified again in vivo. The Cu coating enhanced the original ability of Mg to promote osteogenesis and angiogenesis as well as make the material partly resistant to Staphylococcus aureus.

### 3.3. Mg Alloy

**Figure 1:**
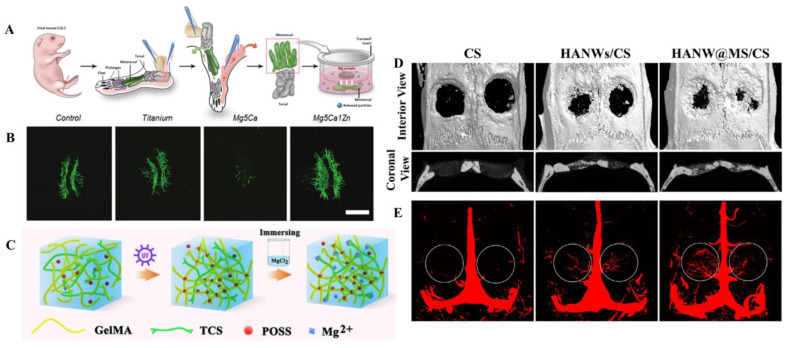
(**A**) Schematic illustration of fetal mouse metatarsal assay culture method © [73]. (**B**) Representative fluorescent images of CD31 positive vessel outgrowth from metatarsal growth under different types of metal alloys © [73]. (**C**) Schematic illustration of the GelMA/TCS/POSS-Mg hydrogel fabricated using the two-step strategy © [76]. (**D**) Micro-CT assessment of newly formed bone and blood vessels in rat calvarial defect regions after implantation of scaffolds of CS, HANWs/CS, and HANW@MS/CS for 12 weeks © [77]. (**E**) Newly formed blood vessels presented by 3D reconstructed images © [77]. Note: CD31: Platelet endothelial cell adhesion molecule-1; GelMA: Gelatin methacrylate; TCS: thiolated chitosan; POSS: polyhedral oligomeric silsesquioxane; Mg: magnesium.

The design of Mg-based alloys is mainly considered from the aspects of enhancing corrosion resistance, reducing hydrogen production, and maintaining biocompatibility. The degradation of Mg alloy implants in vivo is mainly determined by alloying elements. The introduction of most alloying elements such as zinc and aluminum into Mg alloys can improve the oxidation rate, while the introduction of some rare earth elements can reduce the oxidation rate of Mg alloys [67]. Generally, adding manganese (Mn), aluminum (Al), or a small amount of Ca [68] to Mg-based alloys can improve the corrosion resistance [69]. The addition of zinc into Mg-based alloys can increase the yield strength and reduce the production of hydrogen [69]. For the low electrochemical potential of Mg, when Mg alloys are used, they are prone to microgalvanic corrosion of Mg as the anode, and hydrogen gas (H_2_) is generated [70,71]. If used in vivo, the generated H_2_ may cause subcutaneous bubbles and affect surrounding tissues [70]. Alternatively, extrusion can decrease the corrosion rate of Mg due to the average grain size of Mg, which is reduced during the process of extrusion [52,72]. The average grain size of extruded Mg5Ca1Zn (Mg-5 wt%Ca-1 wt%Zn) has been clinically approved [73], and has been refined to ~10 mm by the extrusion [52]. Lee et al. [54] conducted clinical research with a follow-up period of one year. The fractures of the 53 patients healed uneventfully. Additionally, the results of X-ray examination at 6 months and 1 year were consistent with their living animal studies. On this basis, the direct impact of the biodegradation of materials on angiogenesis through a fetal mouse metatarsal assay (Figure 1A) was conducted by Han et al. [73]. This model was used to simulate angiogenesis in vivo; their findings demonstrated that Mg5Ca1Zn could accelerate bone healing by releasing anabolic metallic ions into the surrounding tissues so as to enhance the growth of blood vessels and actively recruit osteoprogenitors near the implant site. Immunofluorescence diagrams (Figure 1B) show that the CD31 positive blood vessels grew from metatarsal bone, and Mg5Ca1Zn had a notable capacity to induce angiogenesis, suggesting that Mg5Ca1Zn has great potential in vascularized osteogenesis for clinical application.

In addition, 6.25% extract liquid of the Mg1Zn2Mn alloy can promote the angiogenesis of HUVEC by activating FGF signal, which may be closely related to the Phosphoinositide 3-kinase (PI3K)/protein kinase B (AKT) pathway [74].

Zhang et al. [75] developed Ca−P-coated Mg−Zn−Gd alloy scaffolds (Ca−P−Mg), which can trigger trigeminal neurons to produce CGRP via transient receptor potential vanilloid subtype 1 (Trpv1); CGRP has been proven as a key factor that promotes angiogenesis and osteogenesis.

Many alloying elements have been applied in Mg-based materials. However, due to the limited solubility of alloying elements in crystalline Mg, the corrosion rate can only be altered in a limited range, and the problem of hydrogen evolution in the degradation process of crystalline Mg alloys remains to be solved [69].

## 4. Metal Materials Releasing Mg Ions

### 4.1. Titanium Alloy

Commercialized pure titanium and titanium alloy (typically Ti6Al4V with 110 GPa Young’s Modulus [69]) are nondegradable materials commonly used in orthopaedics and dentistry (predominantly Ti6Al4V in orthopaedics and pure titanium in dentistry), due to their excellent biocompatibility [78]. In dentistry, an implant bed needs to be drilled into the jaw bone before a titanium dental implant is inserted. Therefore, an artificially created wound is formed which initiates a subsequent series of wound healing processes, and angiogenesis may play a significant role in the osseointegration. Meanwhile, this phenomenon has been demonstrated by the wide genome expression profiling of human peri-implant tissues from 4 days to 2 weeks after implant insertion [79]. Gao et al. [80,81] fabricated Mg-coated Ti6Al4V scaffold using an arc ion plating system in order to ameliorate the characteristics of insufficient bone integration of porous titanium scaffold. The thickness of the Mg coating obtained by their method with fine grain size and high film/substrate adhesion is approximately 5 μm, with a composition of about 1 μm of uniform Mg grains. The in vitro experiment showed that the modified scaffold has the ability to release Mg, with rapid degradation in the first 4 days, which has adverse effects on cells. Fortunately, degradation stabilized after 4 days and promoted cell proliferation. Additionally, Mg-coated Ti6Al4V also displayed favorable osteogenic and angiogenic properties. Moreover, the scaffolds were inserted in rabbit femoral condylar defects. Additionally, the in vivo assay confirmed osteogenesis as well as osseointegration around and inside the scaffold. Microangiography analysis of the 2 mm area around the scaffold showed that, compared to bare Ti6Al4V, more new blood vessels were generated around the Mg-coated Ti6Al4V, indicating that it can accelerate the formation of early blood vessels.

Mg has also been used to enhance the angiogenesis and antibacterial properties of titanium implants. Bacteria around the implant leads to peri-implantitis and failure of osseointegration. Fortunately, some ions such as Cu, Ag, and Zn have strong antibacterial properties [82]. Yu et al. [83] came up with the idea of combining the antibacterial property of Zn with the osteogenic and angiogenic properties of Mg, and prepared a dental implant material—dual Zn/Mg ion co-implanted titanium (Zn/Mg-PIII)—via plasma immersion ion implantation (PIII). The Zn/Mg-PIII can promote the differentiation of rBMSC by exerting the physiological synergy of Zn^2+^ and Mg^2+^; concurrently, it can enhance angiogenesis by promoting the expression of Mg^2+^ transporter 1 (MagT1) in HUVEC, which was proven to be achieved by the releasing of Mg ions.

### 4.2. Tantalum

Tantalum (Ta) has been widely used in the clinical setting because of its biocompatibility and corrosion resistance. However, the elastic modulus of Ta is much higher than that of natural bone (200 vs. 0.1–30 GPa); the stress shielding problem will make the regenerated bone gradually atrophy due to lack of load bearing [84]. 3D printing technology can modify the elastic modulus of the scaffold by changing the porosity and shape of the implant to match that of bone tissues [85], and the porous scaffold has been proven to be able to facilitate vascularized osteogenesis [86]. To sum up, Ma et al. [84] produced 3D porous Ta scaffolds (the elastic modulus of porous Ta was 4.85 ± 0.11 GPa, which is close to that of natural bone) using a selective laser melting (SLM) 3D printer, and then utilizing the surface adhesion ability of polydopamine, doped Mg on the surface of the 3D-printed tantalum scaffolds. The results demonstrate that Ta-PDA-Mg^2+^ can release Mg ions which significantly enhanced the vascularized bone formation and angiogenesis. It is worth noting that the material that can release the highest level of Mg ions (Ta-PDA-Mg^2+^) has the best osteogenic and angiogenic effects in vitro, demonstrating the key role of Mg in promoting vascularized osteogenesis of Mg-enriched biomaterials.

## 5. Mg-Modified Calcium-Phosphate-Based Materials

### 5.1. Mg-Enriched Hydroxyapatite (MHA)

Bone tissue mainly consists of organic collagen and inorganic hydroxyapatite (HA: Ca_10_(PO4)_6_(OH)_2_) with excellent biocompatibility and osteoinductivity [29,87,88]. Since 1970s and 1980s, researchers have performed a series of tests on HA. However, the brickle characteristic and low resorption rate of HA may cause mechanical instabilities and fractures. Therefore, they turned their attention to soluble calcium phosphates (CaP) and created ion-substituted CaP with modified material architecture, and those methods can improve the resorption rate of CaP [89,90]. Partial substitution of HA with Mg results in a nonstoichiometric; this MHA can entirely degrade within 6–12 months. The clinical trials showed that the MHA did not negatively impact the clinical outcome [91,92,93,94]. Doping Mg into porous HA not only enhances the scaffold degradation ability, but also makes the scaffold surface smoother and the micropores more regular, as demonstrated by the study of Deng et al. [90]; in addition, they demonstrated that MHA could enhance the expression level of ALP, Collagen I, and vascular endothelial growth factor (VEGF) in human osteoblasts cells (MG63) through in vitro experiments, though the in vivo angiogenesis level could not be effectively evaluated.

### 5.2. Mg-Enriched CaP Cements/Bioceramics

The research and development of CaP cements (CPC) can be traced back to the early 1980s [95]. Nowadays, CaP bioceramics have been widely used in BTE and clinical applications because they are similar to the composition of bone and they have excellent bioactivity, osteoconductivity, high plasticity, and self-setting properties [96,97,98]. CPC can self-set after mixing with physiological saline or setting solution, and finally produce low-crystallinity HA similar to human hard tissue in structure and composition [99]. However, the disadvantages of bioceramics should not be ignored: bioceramics show rapid ion dissolution and difficult shaping [100].

Mg-phosphate-based cements (MPBC) have a better combination of strength, setting time, and resorption rate, and are regarded as a more ideal bone substitute material than CPC [95]. Wu et al. [98] disclosed citrate regulation on MCPC-mediated angiogenic and osteogenic reactions via a Mg calcium phosphate cement (MCPC) matrix. Their findings proved that MPCP can promote HUVEC polarization and migration to an ordered activation state. Moreover, the HE staining and Masson trichrome staining results of the in vivo experiments showed that MCPC can be gradually absorbed after implantation, and the formation of new bone tissue and blood vessels increased accordingly.

Tricalcium phosphate [Ca_3_(PO_4_)_2_, TCP] has excellent biocompatibility, osteoconductivity, and resorbability. The resorption properties of TCP make it one of the most widely used materials [96]. Mg ions can increase the solubility of phosphate, so Mg phosphate has greater solubility than TCP and HA. Coupled with the special biological functions of Mg ions, Mg-phosphate-based materials have greater superiorities than TCP and HA as bone substitutes [88]. Bose et al. [101] used 3D printing technology to fabricate TCP scaffolds with different interconnected macropore sizes. These printed TCP scaffolds showed huge potential for bone tissue repair and regeneration due to the continuously released Mg and Si ions, though the increase in angiogenesis was attributed to pore interconnectivity and multiscale porosity in these TCP scaffolds.

Silicocarnotite [Ca_5_(PO_4_)_2_SiO_4_, CPS] has the potential to be a desirable bone repair material because it has better cytocompatibility and solubility than conventional CaP such as HA [102]. Wu et al. [103] designed and fabricated Mg-silicocarnotite [Mg-Ca_5_(PO_4_)_2_SiO_4_, Mg-CPS], demonstrating that the introduction of MgO can improve the osteogenesis and angiogenesis of CPS. The osteogenic differentiation of MC3T3-E1 and angiogenic effect of HUVEC were significantly enhanced when the incorporation of MgO ranged from 0 wt% to 10 wt%. Moreover, the mechanisms of the aforementioned phenomenon could be attributed to the activating of the Smad2/3/-Runx2 pathway in MC3T3-E1 cells and the PI3K/AKT signal pathway in HUVEC trigged by the released Mg ions.

## 6. New Class of Biomaterial

Clay nanoparticles, high polymers, hydrogels, and composites are new kinds of biomaterials which have great potential in BTE [104].

### 6.1. Mg-Enriched Biodegradable Polymer

As a biodegradable polymer, Poly(lactic-co-glycolic) acid (PLGA) has been used as a clinical bone repair material due to its degradability and adjustable biocompatibility [105]. PLGA-based products have been approved by the Food and Drug Administration (FDA) for biomedical usage, among which OsteoScaf™ (TRT, Toronto, ON, Canada) has been used clinically [105,106]. However, the low mechanical properties and local acidification of PLGA often lead to the failure of its clinical application [107]. By coating Mg hydroxide and bone-extracellular matrix (ECM) on porous PLGA scaffolds, Kim et al. [107] fabricated PMEP scaffolds with the bioactive polydeoxyribonucleotide added into these PME scaffolds, which could inhibit osteoclastogenesis while promoting adequate cell proliferation, angiogenesis, and osteogenesis in vitro. Lai et al. [1] used Mg powder, PLGA, and β-tricalcium phosphate (β-TCP) elements to engineer a novel porous PLGA/TCP/Mg (PTM) scaffold by low-temperature rapid prototyping technology (LT-RP). The angiogenesis and osteogenesis effects of the PTM scaffolds were evaluated in established steroid associated osteonecrosis (SAON) rabbit models. The in vivo experiments revealed that this modified scaffold can provide space for vascular crawling, resulting in neo-angiogenesis formation, which finally facilitated the formation and remodeling of new bone.

Guo et al. [108] found that Mg^2+^ can promote the osteogenesis of MC3T3-E1 and angiogenesis of HUVEC by up-regulating the secretion of Platelet-derived growth factor-BB (PDGF-BB) from the former in their previous in vitro experiments. Based on the results of this research, they fabricated a Polyetheretherketone (PEEK)-based material via 3D printing technology. The elastic modulus of PEEK was revised to be close to that of human cancellous bone. Then, the bioactivity of the printed PEEK was further enhanced by coating it with a Poly-dopamine (PDA) coating and Mg^2+^ [109].

Polybutylene succinate (PS) is an aliphatic thermoplastic polyester with biodegradable, nontoxic, and biocompatible properties. However, the sluggish degradation rate makes it undesirable for bone regeneration [110]. Zhao et al. [110] accelerated the degradation rate and bioactivity of the PS by adding Mg phosphate (40 wt%) and wheat protein (10 wt%) to it. The composited material PMWC significantly promoted the proliferation and differentiation of MC3T3-E1 in vitro, and also bolstered osteogenesis and angiogenesis in vivo.

### 6.2. Mg-Enriched Hydrogels

Hydrogel is a kind of water-swollen polymeric biomaterial composed of a 3D hydrophilic network with an abundance of water. Traditional hydrogel synthesis methods include crosslinking copolymerization, the crosslinking of reactive polymer precursors, and crosslinking via polymer–polymer reaction [111,112]. Hydrogels can mimic ECM as its structure is similar to the macromolecular-based components in the body [113]. When designing tissue-engineering hydrogels, the following aspects should be taken into consideration: mechanical properties, controlled degradation, and the interaction between cells and hydrogels [113].

#### 6.2.1. Hydrogels from Synthetic Polymers

Chen et al. [114] uniformly mounted osteoconductive HA nanocrystals and osteoinductive magnesium oxide (MgO) nanocrystals into the network matrix of an organic hydrogel composed of cysteine-modified γ-polyglutamic acid (PGA-Cys) to construct a hydrogel scaffold (HA/MgO-H). This hybrid hydrogel (Young’s modulus 0.78 ± 0.17 MPa) can reduce proinflammatory macrophage infiltration and improve angiogenesis (CD31^+^) in order to provide a favorable microenvironment for bone repair in rats with type Ⅰ diabetes mellitus. The in vitro results indicated that Mg^2+^ enhanced the expression level of Col-Ⅰ, the main component of ECM, though the Mg^2+^ seemed to have no effect on CD31^+^ neovascularization.

#### 6.2.2. Gelatin Methacrylate

A Gelatin with methacryloyl side groups—Gelatin methacrylate (GelMA)—is a photosensitive hydrogel material that has been widely used as 3D scaffolds [115]. GelMA can mimic the structure of ECM, but it has no osteoinduction to induce bone formation and needs to be combined with other bioactive materials to endow it with osteogenic effects [76,116].

Luo et al. [117] made PLGA microspheres loaded with La_2_(CO_3_)_3_, and embedded it into a MgO/MgCO_3_-loaded cryogel made of photocrosslinkable GelMA to enable the co-delivery of Mg^2+^ and La^3+^. This co-delivery system of Mg^2+^ and La^3+^ had a synergistic effect in promoting vascularized bone formation in their study.

In order to improve the mechanical performance of hydrogels and stimulate local bone healing by using Mg^2+^ to promote neo-angiogenesis, Zhang et al. [76] prepared a Mg ion-incorporating dual-crosslinked hydrogel via a two-step method of photopolymerization and Mg–S coordination (Figure 1C). The photocrosslinking characteristics of GelMA were adopted, polyhedral oligomeric silsesquioxane (POSS) was employed to strengthen the polymer grid structure, and Mg^2+^ ions were then introduced into the system via coordination bonds of Mg–S; thiolated chitosan (TCS) was further used to bridge Mg^2+^, by which a new hydrogel network system was finally established. This modified hydrogel possesses enhanced mechanical properties and advocates a Mg-enriched microenvironment, expediting the osteogenesis of stem cells and angiogenesis of endothelial cells. The Young’s modulus of this material was significantly higher than GelMA, but still could not match that of natural bone.

Interestingly, in the study by Luo et al. [118], when black phosphorus (BP) nanosheets were added to GelMA, the elastic modulus of the material significantly increased. However, when Mg-modified BP (BP@Mg) was added to GelMA, there was no significant change in the elastic modulus of the material compared to the group only incorporated with BP nanosheets. The change in swelling rate was also inapparent, indicating that Mg may not necessarily have a significant impact on the elastic modulus. In their previous study, they added this BP@Mg to GELMA as a bionic periosteum structure and found that it can promote angiogenesis [119]. The upper bionic periosteal structure and the lower GelMA-PEG/β-TCP hydrogel together forms a double-layer hydrogel scaffold, and Mg can enhance the osteogenic properties of the scaffold. In in vivo experiments, the early angiogenesis and bone formation in the surrounding bone defect area of the Mg-free double-layer hydrogel group were less than those of the Mg-containing group.

#### 6.2.3. Injectable Hydrogel

Scaffold implantation requires a large enough incision for the insertion of the scaffold. On the other hand, injectable hydrogel is an excellent scaffold material for the regeneration of irregular non-load-bearing bone defects [120]. Mg-enriched injectable hydrogels usually have a basic injectable hydrogel system, and then Mg or Mg-containing materials are incorporated into the system. For instance, Jiang’s group [121] fabricated a three-dimensional (3D) culture system based on the incorporation of Mg ammonium phosphate hexahydrate (struvite) into GelMA, which advanced the osteoinduction of GelMA successfully. This novel injectable composite hydrogel can release the ionic components in vitro, promoting the osteogenic differentiation of dental pulp stem cells (DPSCs) and enhancing the chemoattraction of HUVEC to increase angiogenesis. Similarly, Han et al. [122] made an injectable SAG hydrogel comprising of sodium alginate, akermanite (Aker), and glutamic acid which could be used for wound healing. Zhang’s group [100] noticed the value of injectable SAG hydrogel systems in bone regeneration. They examined the osteogenic effect of SAG composite injectable hydrogel in vitro and in vivo. The results showed that the hydrogel extract induced osteogenesis in a concentration-dependent manner, and the ideal concentration was regarded as 66.9 ppm for Ca^2+^, 23.8 ppm for Mg^2+^, and 33.5 ppm for Si^2+^.

In order to further enhance the angiogenic potential of the material, Priya et al. [120] added Mg-doped bioglass (MBG) and fibrin nanoparticles (FNPs, 250 ± 20 nm) to the basic injectable hydrogel system made of chitin and poly (butylene succinate) (PBSu). Of note, fibrin was essential for the formation of microvascular networks and Col Ⅰ. The properties of the above-mentioned material showed that the addition of MBG increased the elastic modulus of the hydrogels, while the addition of FNPs had an inverse effect due to the hydrophilic nature of fibrin. In addition, after optimizing the proportion of additives, it was finally concluded that the hydrogel system with 2% MBG and 2% FNPs exhibited angiogenic as well as osteogenic potential; it also showed desirable injectability and temperature stability ranging from 25 to 45 °C. Therefore, this injectable hydrogel sheds light on the treatment for irregular bone defects.

Furthermore, there are several innovative injectable hydrogels. Tang et al. [123] focused on the emission of H_2_ gas during the degradation of Mg. They employed Mg particles (MPs) as foaming agents and incorporated these particles into hydrogel solutions with living cells incubated in it. These injectable microporous hydrogels possessed injectable, porous, and biocompatible characteristics, but the elastic modulus inevitably decreased due to the increased porosity of the porous hydrogel. They believe that this material could form a mild alkaline environment and alleviate acidosis caused by material implantation, which further promoted the generation of intact vascularized bone in three weeks. Jiang’s group [6] utilized the coordinative reaction between metal ion ligands to construct a bisphosphonate functionalized injectable hydrogel microsphere (GelMA-BP-Mg). Instead of introducing Mg ions, the hydrogel microsphere captured Mg^2+^ in the body; the atomic percentage of captured Mg^2+^ was 0.6%, which could be continuously released for 18 days. Moreover, this hydrogel promoted bone formation and angiogenesis by stimulating osteoblasts and endothelial cells while inhibiting osteoclasts. Table 2 summarizes the characteristics and functions of Mg-enriched injectable hydrogels.

### 6.3. Clay Nanoparticles

Clay has a high retention capacity and swelling and rheological properties. Clay minerals are a family of inorganic layered nanomaterials which can enhance the mechanical and degradation properties of polymer matrixes and are increasingly used in biomedicine [104]. Taking advantage of the nontoxic and bioactive characteristics of natural attapulgite (ATP, structural formula (Al_2_Mg_2_)Si_8_O_2_0(OH)_2_(OH_2_)_4_·4H_2_O) nanorods, Wang et al. [124] used a 3D bioprinter to manufacture a novel porous nano-ATP scaffold and bonded it with polyvinyl alcohol as a binder, then sintered it to obtain the end-product. This scaffold could directly induce bone formation by membranous ossification and promote the revascularization of the defect zone in a rat cranium defect model. In vitro experiments have also shown that it has good biocompatibility while promoting the osteogenesis of human BMSCs.

### 6.4. Nanomaterials

Chen et al. [77] have previously synthesized a highly flexible HA wire using calcium oleate as a precursor. With the foundation in front, they used HA wire and chitosan (CS) to simulate inorganic components and collagen of bone, respectively. Thereafter, they synthesized HANW@MS core−shell porous hierarchical nanobrushes composed of hydroxyapatite nanowires (HANWs) as the core and Mg silicate nanosheets (MS) as the shell, and used it to fabricate HANW@MS/CS scaffolds (size 15 × 10 mm). These HANW@MS/CS scaffolds can release Mg^2+^ and SiO_3_^2−^ in vitro, promoting the osteogenic differentiation of rBMSC and simulating the formation of new bone and neovascularization. The results of in vivo assays showed that the scaffolds were capable of releasing Mg and Si ions, both of which further boosted new bones and blood vessel formation in the defect area [77] (Figure 1D,E).

## 7. Mechanism Research

### 7.1. CGRP-Mediated Pathway

**Figure 2:**
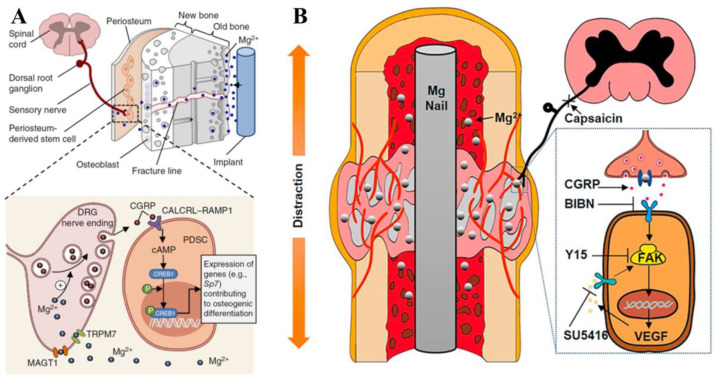
(**A**) Schematic diagram of how implant-derived magnesium induces local neuronal production of calcitonin gene-related peptide (CGRP) to promote the expression of genes contributing to osteogenic differentiation © [59]. (**B**) Schematic diagram showing the proposed mechanism of pure Mg nail-enhanced critical-size bong defect repair during distraction osteogenesis (DO) © [57].

CGRP is a neuropeptide related to bone healing which can be released from the sensory neuronal endings in the periosteum of the long-bone shaft [59]. Qin et al. [59] proved that the Mg ions released from the implant promoted the synthesis of CGRP in the dorsal root ganglion (DRG) and the sensory nerve rooted in periosteum as well. Because Mg ions can enter into neurons mediated by MAGT1 and transient receptor potential melastatin 7 (TRPM7), the intracellular Mg ions promote actin polymerization and further facilitate the aggregation of CGRP vesicles at the synapses [59] (Figure 2A). Since then, the CGRP-mediated pathway has been identified as the main mechanism of Mg-promoting bone formation in the process of fracture healing. Their study also found that CGRP or Mg implants did not up-regulate the expression of Runx2, a key osteogenic gene. Therefore, other regulatory pathways may also be involved in the osteogenic differentiation regulatory effects of Mg ions. Their subsequent experiment [57] on DO found that CGRP increased angiogenesis through the focal adhesion kinase (FAK)-VEGF signal pathway, which may be the key mechanism of Mg accelerating the DO process, showing the potential of Mg in the application of DO therapy (Figure 2B). However, Hamushan et al. [60] found that Mg may enhance angiogenesis and bone consolidation via the regulation of the Von Hippel–Lindau (VHL)/hypoxia-inducible factor-1α (HIF-1α)/VEGF pathway.

### 7.2. Pro-Osteogenic Immune Microenvironment

Wang et al. [97] prepared a magnesium calcium phosphate cement (MCPC) by mixing CPC and magnesium phosphate cement (MPC) powder together with deionized water (90 wt% CPC, 10 wt% CPC, 0.3 mL g-1deionized water). The in vitro studies showed that the MPCP could transform macrophage responses from the M1 phenotype to M2 phenotype, and this immune regulation was conducive to the osteogenesis of BMSC and the angiogenesis of HUVEC. Since the Ca^2+^ released by MCPC in vitro was similar to that of CPC, this immunoregulatory effect was mainly attributed to the release of Mg^2+^.

### 7.3. PI3K/AKT Pathway Signals

The results of some studies show that the method by which Mg-enriched materials promote vascularized osteogenesis may be closely related to the PI3K/AKT signaling pathway [74].

**Figure 3:**
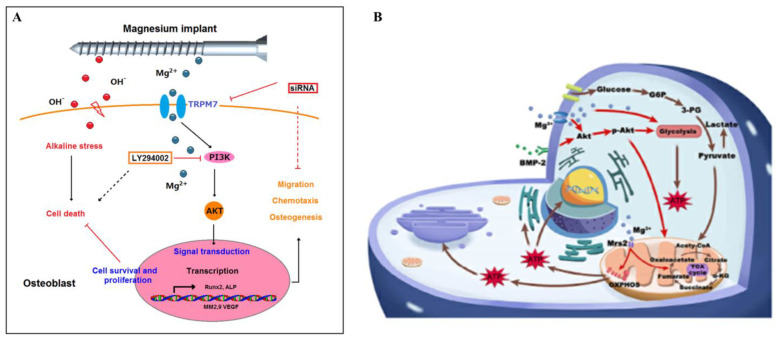
(**A**) A proposed model for how Mg-ion-mediated TRPM7/PI3K pathway signals confer osteogenesis and resistance to alkaline-stress-induced cytotoxicity in human osteoblasts © [127]. (**B**) Mechanism of the stimulatory effects of Mg^2+^ on the efficacy of BMP-2 © [128]. Note: TRPM7: transient receptor potential melastatin 7; PI3K: phosphoinositide 3-kinase; BMP-2: bone morphogenetic protein-2.

The degradation of the Mg-based material after implantation creates a local alkaline environment peripherally, which is a double-edged sword. On the one hand, the alkaline environment can promote osteoblast proliferation; on the other hand, a high-alkaline environment can force osteosarcoma cells into dormancy. Thus, the search for the mechanism of Mg action in these cases is of great significance for both osteosarcoma therapy and the production of orthopaedic implants [125,126]. Zhang et al. [127] found that the effective concentration of Mg ions (5 × 10^−3^ mol/L–10 × 10^−3^ mol/L) could activate PI3K phosphorylation via Mg ion transporter TRPM7, which triggered hFOB1.19 (human osteoblast cell) recruitment, osteogenesis, and resistance to alkaline stress. Additionally, the expression of VEGF in osteoblasts increased significantly after the treatment of Mg with the concentration of less than 5 ×10^−3^ mol/L. Their research provided a new view of Mg-enriched materials in the treatment of osteosarcoma: targeting the activity of the TRPM7 and PI3K pathways (Figure 3A).

Recently, Lin et al. [128] found that bone morphogenetic protein-2 (BMP-2) boosted the osteogenic differentiation of BMSC by promoting AKT-pathway-related metabolic reprogramming in a dose-dependent manner. Additionally, the low concentration of Mg-rich environment (5 × 10^−3^ mol/L) enhanced the osteogenic response to low doses of BMP-2 (20 μg/mL). In the subcutaneous ectopic bone formation mouse model, the Mg-rich environment could considerably promote the formation of vascularized new bone (Figure 3B). The mechanisms of Mg-enriched materials promoting vascularized osteogenesis are listed in Table 3.

## 8. Conclusions and Prospects

Although many studies have shown that Mg-enriched materials have excellent osteogenic ability, it has also been observed that Mg has adverse effect on osteogenesis after fast degradation [63]. Therefore, it is still necessary to clarify the mechanism of Mg-promoting bone healing.

At present, Mg-enriched materials are rarely used as bone repair materials in clinics. The main reason is the degradation of Mg-enriched materials in human body is difficult to track and the local ion concentration cannot be measured. The degradation process can only be predicted through in vitro degradation experiments and animal models. If a more effective prediction model can be created, it will promote the safer, more reasonable, and more controllable application of Mg-enriched materials in clinical surgery [73]. All in all, these in vitro and in vivo studies are very different from the complicated physiological environment in the human body.

For now, in vitro experiments of vascularized osteogenesis mainly use HUVEC to verify the correlation with blood vessels. However, this cell line is derived from fetal umbilical cords and lacks adult cellular markers [129]. In the future, the source of endothelial cells should be considered when designing experiments, so that their phenotypes match the organotypic features [129]. Moreover, more models that can dynamically observe angiogenesis and osteogenesis should be explored. The fetal mouse metatarsal bone model is a relatively fine and time-consuming unique tool for studying angiogenesis [130]. However, few uses of this model have been seen in the study of angiogenesis and osteogenesis. More use of this model in future experiments may improve the credibility of the research results.

Angiogenesis is regulated by two pathways, one is the VEGF-dependent pathway and the other is the angiopoietin-dependent pathway [131], both of which may be related to the process of bone repair. At present, there are few studies on the latter in the research of Mg-rich materials promoting angiogenesis; this can be studied in the future.

Mg-enriched materials for vascularized osteogenesis have been studied in many bone defect models, such as the distraction osteogenesis model [57,60] and the orbital bone defect model [75]. In the future, more research can expand on the use of Mg-enriched materials in more bone defect models.

## Figures and Tables

**Table 1:** Mechanical properties of magnesium (Mg) -based materials for clinical use compared to cancellous and cortical bone.

Tissue/Materials	Density (g/cm^3^)	Young’s Module (GPa)	Yield Strength (MPa)	Compression Strength (MPa)	Tensile Strength (MPa)	Fatigue Strength (MPa, 10^7^ Cycles)	Author/Year
Cortical bone	1.8–2.0	7–30	NA	100–230	164–240	27–35	Zhao, D./2017 [4], Dragosloveanu, S./2021 [35]
Cancellous bone	1.0–1.4	0.01–3.0	NA	2–12	NA	NA	Zhao, D./2017 [4]
Pure Mg (99.9%, casted)	1.74	41	21	40	87	NA	Zhao, D./2017 [4], Staiger, M.P./2006 [18]
Pure Mg (99.9%, wrought)	1.74	41	100	100–140	180	NA	Zhao, D./2017 [4], Staiger, M.P./2006 [18]
MgYReZr	1.84	45	235	NA	Above 275	NA	Zhao, D./2017 [4], Dragosloveanu, S./2021 [35], Ezechieli, M./2016 [36], Sontgen, S./2023 [37]
Mg-Ca-Zn	1.80	NA	NA	415	249	NA	Cho, S. Y./2012 [38]

Note: NA: not available.

**Table 2:** Characteristics and functions of Mg-enriched injectable hydrogels.

Materials	Characteristic	Experiments	Animal Model	Functions	Author/ Year
Struvite Composite Cell-Laden Hydrogel	elastic modulus: approximately 7.26 × 10^3^ Pa	in vitro	-	GelMA: has fluidity, stability, and degradability Composite: promotes osteogenesis and angiogenesis	Liu, C./2021 [121]
Chitin-PBSu hydrogel system with 2%MBG and 2%FNPs	elastic modulus: approximately 1.45 × 10^5^ Pa	in vitro	-	chitin-PBSu hydrogel: mimics the ECM; provides cues for the surrounding cells to proliferate; helps in healing the defect site FNPs: enhances the cell attachment and spreading; angiogenic property MBG: promotes higher protein adsorption for helping in better cell attachment and spreading; possess osteoinductive and angiogenic properties	Vishnu Priya, M./2016 [120]
SAG hydrogel	the pore size ranged of freeze-dried porous scaffolds from 150 to 250 μm	in vivo	maxillary sinus floor elevation in rabbits	promotes bone formation via CXCR4 elevation and ERK signaling pathway	Zhang, X./2018 [100]
injectable macroporous hydrogels	void ratio 73.04 ± 5.92%	in vivo	SD rat femur defects model	Mg-degradation-dependent H_2_-foaming method directly generated pores in cell-laden hydrogels while sustaining the injectability and cytocompatibility of the hydrogels	Tang, Y./2020 [123]

Note: GelMA: Gelatin methacrylate; PBSu: poly (butylene succinate); ECM: extracellular matrix; FNP: fibrin nanoparticles; MBG: magnesium-doped bioglass; CXCR4: C-X-C chemokine receptor type 4; ERK: extracellular regulated protein kinases; SD: Sprague Dawley.

**Table 3:** Mechanisms of Mg-enriched materials promoting vascularized osteogenesis.

Materials	Characteristic	Experiments	Animal Model	Angiogenesis Mechanism	Author/Year
Mg nail	Purity of 99.99%	in vivo	Critical size midshaft femur bone defect (5 mm in length) model	up-regulated the expression of CGRP, CGRP promoted the phosphorylation of FAK and elevated the expression of VEGFA	Ye, L./2021 [57]
High-purity Mg pin	Length of 5 mm and diameter of 1 mm	in vivo	rat distraction osteogenesis model	via the regulation of VHL/HIF-1α/VEGF signaling	Hamushan, M./2020 [60]
MCPC	Contain CPC powder, MPC powder and liquid phase (deionized water)	in vitro	-	Regulation of HUVEC angiogenesis in vitro by immune regulation of macrophages	Wang, M./2016 [97]
microgel composite hydrogels	BMP-2/Mg^2+^ codelivery platform	in vivo	critical cranial defect mode	increase cellular bioenergetic levels to fuel osteogenesis, and thereby markedly promoted the osteoinductivity of BMP-2.	Lin, S./2022 [128]

Note: CGRP: calcitonin gene-related peptide; FAK: Focal Adhesion Kinase; VEGF: vascular endothelial growth factor; VHL: Von Hippel–Lindau; HIF-1α: hypoxia-inducible factor-1α; CPC: calcium phosphate cements; MPC: magnesium phosphate cement; HUVEC: human umbilical vein endothelial cell; BMP-2: bone morphogenetic protein-2.

## Data Availability

Not applicable.

## References

[1] Lai Y., Li Y., Cao H., Long J., Wang X., Li L., Li C., Jia Q., Teng B., Tang T. (2019). Osteogenic magnesium incorporated into PLGA/TCP porous scaffold by 3D printing for repairing challenging bone defect. Biomaterials.

[2] Peng Z., Zhao T., Zhou Y., Li S., Li J., Leblanc R.M. (2020). Bone Tissue Engineering via Carbon-Based Nanomaterials. Adv. Healthc. Mater..

[3] Wang J.L., Xu J.K., Hopkins C., Chow D.H., Qin L. (2020). Biodegradable Magnesium-Based Implants in Orthopedics-A General Review and Perspectives. Adv. Sci..

[4] Zhao D., Witte F., Lu F., Wang J., Li J., Qin L. (2017). Current status on clinical applications of magnesium-based orthopaedic implants: A review from clinical translational perspective. Biomaterials.

[5] Li Y., Jahr H., Zhou J., Zadpoor A.A. (2020). Additively manufactured biodegradable porous metals. Acta Biomater..

[6] Zhao Z., Li G., Ruan H., Chen K., Cai Z., Lu G., Li R., Deng L., Cai M., Cui W. (2021). Capturing Magnesium Ions via Microfluidic Hydrogel Microspheres for Promoting Cancellous Bone Regeneration. ACS Nano.

[7] Anada T., Pan C.C., Stahl A.M., Mori S., Fukuda J., Suzuki O., Yang Y. (2019). Vascularized Bone-Mimetic Hydrogel Constructs by 3D Bioprinting to Promote Osteogenesis and Angiogenesis. Int. J. Mol. Sci..

[8] Glowacki J. (1998). Angiogenesis in fracture repair. Clin. Orthop. Relat. Res..

[9] Santos M.I., Reis R.L. (2010). Vascularization in bone tissue engineering: Physiology, current strategies, major hurdles and future challenges. Macromol. Biosci..

[10] Simunovic F., Finkenzeller G. (2021). Vascularization Strategies in Bone Tissue Engineering. Cells.

[11] Stegen S., van Gastel N., Carmeliet G. (2015). Bringing new life to damaged bone: The importance of angiogenesis in bone repair and regeneration. Bone.

[12] Albaraghtheh T., Willumeit-Römer R., Zeller-Plumhoff B. (2022). In silico studies of magnesium-based implants: A review of the current stage and challenges. J. Magnes. Alloys.

[13] Li D., Yuan Y., Liu J., Fichtner M., Pan F. (2020). A review on current anode materials for rechargeable Mg batteries. J. Magnes. Alloys.

[14] Liu B., Yang J., Zhang X., Yang Q., Zhang J., Li X. (2023). Development and application of magnesium alloy parts for automotive OEMs: A review. J. Magnes. Alloys.

[15] Sun J., Du W., Fu J., Liu K., Li S., Wang Z., Liang H. (2022). A review on magnesium alloys for application of degradable fracturing tools. J. Magnes. Alloys.

[16] Vijaya Ramnath B., Kumaran D., Melvin Antony J., Rama Subramanian M., Venkatram S., Mazlan N., Sapuan S.M., Ilyas R.A. (2022). Studies on Magnesium Alloy: Composites for Aerospace Structural Applications. Advanced Composites in Aerospace Engineering Applications.

[17] Walker J., Shadanbaz S., Woodfield T.B., Staiger M.P., Dias G.J. (2014). Magnesium biomaterials for orthopedic application: A review from a biological perspective. J. Biomed. Mater. Res. B Appl. Biomater..

[18] Staiger M.P., Pietak A.M., Huadmai J., Dias G. (2006). Magnesium and its alloys as orthopedic biomaterials: A review. Biomaterials.

[19] Song J., She J., Chen D., Pan F. (2020). Latest research advances on magnesium and magnesium alloys worldwide. J. Magnes. Alloys.

[20] Šalandová M., van Hengel I.A.J., Apachitei I., Zadpoor A.A., van der Eerden B.C.J., Fratila-Apachitei L.E. (2021). Inorganic Agents for Enhanced Angiogenesis of Orthopedic Biomaterials. Adv. Healthc. Mater..

[21] Bennett J., De Hemptinne Q., McCutcheon K. (2019). Magmaris resorbable magnesium scaffold for the treatment of coronary heart disease: Overview of its safety and efficacy. Expert. Rev. Med. Devices.

[22] Nasr Azadani M., Zahedi A., Bowoto O.K., Oladapo B.I. (2022). A review of current challenges and prospects of magnesium and its alloy for bone implant applications. Prog. Biomater..

[23] Nie X., Zhang X., Lei B., Shi Y., Yang J. (2022). Regulation of Magnesium Matrix Composites Materials on Bone Immune Microenvironment and Osteogenic Mechanism. Front. Bioeng. Biotechnol..

[24] Shan Z., Xie X., Wu X., Zhuang S., Zhang C. (2022). Development of degradable magnesium-based metal implants and their function in promoting bone metabolism (A review). J. Orthop. Translat..

[25] Wlodarczak A., Montorsi P., Torzewski J., Bennett J., Starmer G., Buck T., Haude M., Moccetti M., Wiemer M., Lee M.K. (2023). One- and two-year clinical outcomes of treatment with resorbable magnesium scaffolds for coronary artery disease: The prospective, international, multicentre BIOSOLVE-IV registry. EuroIntervention.

[26] Zong J., He Q., Liu Y., Qiu M., Wu J., Hu B. (2022). Advances in the development of biodegradable coronary stents: A translational perspective. Mater. Today Bio..

[27] Zhao N., Zhu D. (2015). Endothelial responses of magnesium and other alloying elements in magnesium-based stent materials. Metallomics.

[28] Ji B., Gao H. (2004). Mechanical properties of nanostructure of biological materials. J. Mech. Phys. Solids.

[29] Kim H.D., Park J., Amirthalingam S., Jayakumar R., Hwang N.S. (2020). Bioinspired inorganic nanoparticles and vascular factor microenvironment directed neo-bone formation. Biomater. Sci..

[30] Lin S., Yang G., Jiang F., Zhou M., Yin S., Tang Y., Tang T., Zhang Z., Zhang W., Jiang X. (2019). A Magnesium-Enriched 3D Culture System that Mimics the Bone Development Microenvironment for Vascularized Bone Regeneration. Adv. Sci..

[31] Witte F. (2010). The history of biodegradable magnesium implants: A review. Acta Biomater..

[32] Biber R., Pauser J., Brem M., Bail H.J. (2017). Bioabsorbable metal screws in traumatology: A promising innovation. Trauma. Case Rep..

[33] Notice of Clinical Trial Approval Issued on 10 July 2019. https://www.nmpa.gov.cn/directory/web/nmpa/zwfw/sdxx/sdxxylqx/qxpjfb/20190710105301598.html.

[34] Zhao D., Huang S., Lu F., Wang B., Yang L., Qin L., Yang K., Li Y., Li W., Wang W. (2016). Vascularized bone grafting fixed by biodegradable magnesium screw for treating osteonecrosis of the femoral head. Biomaterials.

[35] Dragosloveanu S., Cotor D.C., Dragosloveanu C.D.M., Stoian C., Stoica C.I. (2021). Preclinical study analysis of massive magnesium alloy graft for calcaneal fractures. Exp. Ther. Med..

[36] Ezechieli M., Meyer H., Lucas A., Helmecke P., Becher C., Calliess T., Windhagen H., Ettinger M. (2016). Biomechanical Properties of a Novel Biodegradable Magnesium-Based Interference Screw. Orthop. Rev..

[37] Sontgen S., Keilig L., Kabir K., Weber A., Reimann S., Welle K., Bourauel C. (2023). Mechanical and numerical investigations of biodegradable magnesium alloy screws for fracture treatment. J. Biomed. Mater. Res. B Appl. Biomater..

[38] Cho S.Y., Chae S.W., Choi K.W., Seok H.K., Han H.S., Yang S.J., Kim Y.Y., Kim J.T., Jung J.Y., Assad M. (2012). Load-bearing capacity and biological allowable limit of biodegradable metal based on degradation rate in vivo. J. Biomed. Mater. Res. B Appl. Biomater..

[39] Windhagen H., Radtke K., Weizbauer A., Diekmann J., Noll Y., Kreimeyer U., Schavan R., Stukenborg-Colsman C., Waizy H. (2013). Biodegradable magnesium-based screw clinically equivalent to titanium screw in hallux valgus surgery: Short term results of the first prospective, randomized, controlled clinical pilot study. Biomed. Eng. Online.

[40] Waizy H., Diekmann J., Weizbauer A., Reifenrath J., Bartsch I., Neubert V., Schavan R., Windhagen H. (2014). In vivo study of a biodegradable orthopedic screw (MgYREZr-alloy) in a rabbit model for up to 12 months. J. Biomater. Appl..

[41] Ezechieli M., Diekmann J., Weizbauer A., Becher C., Willbold E., Helmecke P., Lucas A., Schavan R., Windhagen H. (2014). Biodegradation of a magnesium alloy implant in the intercondylar femoral notch showed an appropriate response to the synovial membrane in a rabbit model in vivo. J. Biomater. Appl..

[42] Diekmann J., Bauer S., Weizbauer A., Willbold E., Windhagen H., Helmecke P., Lucas A., Reifenrath J., Nolte I., Ezechieli M. (2016). Examination of a biodegradable magnesium screw for the reconstruction of the anterior cruciate ligament: A pilot in vivo study in rabbits. Mater. Sci. Eng. C Mater. Biol. Appl..

[43] Ezechieli M., Ettinger M., Konig C., Weizbauer A., Helmecke P., Schavan R., Lucas A., Windhagen H., Becher C. (2016). Biomechanical characteristics of bioabsorbable magnesium-based (MgYREZr-alloy) interference screws with different threads. Knee Surg. Sports Traumatol. Arthrosc..

[44] Hagelstein S., Seidenstuecker M., Kovacs A., Barkhoff R., Zankovic S. (2022). Fixation Performance of Bioabsorbable Zn-6Ag Pins for Osteosynthesis. Materials.

[45] Acar B., Kose O., Unal M., Turan A., Kati Y.A., Guler F. (2020). Comparison of magnesium versus titanium screw fixation for biplane chevron medial malleolar osteotomy in the treatment of osteochondral lesions of the talus. Eur. J. Orthop. Surg. Traumatol..

[46] Biber R., Pauser J., Geßlein M., Bail H.J. (2016). Magnesium-Based Absorbable Metal Screws for Intra-Articular Fracture Fixation. Case Rep. Orthop..

[47] Kose O., Turan A., Unal M., Acar B., Guler F. (2018). Fixation of medial malleolar fractures with magnesium bioabsorbable headless compression screws: Short-term clinical and radiological outcomes in eleven patients. Arch. Orthop. Trauma. Surg..

[48] Kozakiewicz M. (2021). Change in Pull-Out Force during Resorption of Magnesium Compression Screws for Osteosynthesis of Mandibular Condylar Fractures. Materials.

[49] Turan A., Kati Y.A., Acar B., Kose O. (2020). Magnesium Bioabsorbable Screw Fixation of Radial Styloid Fractures: Case Report. J. Wrist Surg..

[50] Ünal M., Demirayak E., Ertan M.B., Kilicaslan O.F., Kose O. (2022). Bioabsorbable magnesium screw fixation for tibial tubercle osteotomy; a preliminary study. Acta Biomed..

[51] Meier R., Panzica M. (2017). First results with a resorbable MgYREZr compression screw in unstable scaphoid fractures show extensive bone cysts. Handchir. Mikrochir. Plast. Chir..

[52] Cha P.R., Han H.S., Yang G.F., Kim Y.C., Hong K.H., Lee S.C., Jung J.Y., Ahn J.P., Kim Y.Y., Cho S.Y. (2013). Biodegradability engineering of biodegradable Mg alloys: Tailoring the electrochemical properties and microstructure of constituent phases. Sci. Rep..

[53] Cho S.Y., Chae S.W., Choi K.W., Seok H.K., Kim Y.C., Jung J.Y., Yang S.J., Kwon G.J., Kim J.T., Assad M. (2013). Biocompatibility and strength retention of biodegradable Mg-Ca-Zn alloy bone implants. J. Biomed. Mater. Res. B Appl. Biomater..

[54] Lee J.W., Han H.S., Han K.J., Park J., Jeon H., Ok M.R., Seok H.K., Ahn J.P., Lee K.E., Lee D.H. (2016). Long-term clinical study and multiscale analysis of in vivo biodegradation mechanism of Mg alloy. Proc. Natl. Acad. Sci. USA.

[55] Sharma S., Abhishekrathi, Mittal G., Ranjan R. (2016). Distraction Osteogenesis—Evolution & Technique- An Overview. J. Dent. Med..

[56] Ai-Aql Z.S., Alagl A.S., Graves D.T., Gerstenfeld L.C., Einhorn T.A. (2008). Molecular mechanisms controlling bone formation during fracture healing and distraction osteogenesis. J. Dent. Res..

[57] Ye L., Xu J., Mi J., He X., Pan Q., Zheng L., Zu H., Chen Z., Dai B., Li X. (2021). Biodegradable magnesium combined with distraction osteogenesis synergistically stimulates bone tissue regeneration via CGRP-FAK-VEGF signaling axis. Biomaterials.

[58] Ilizarov G.A. (1989). The tension-stress effect on the genesis and growth of tissues. Part I. The influence of stability of fixation and soft-tissue preservation. Clin. Orthop. Relat. Res..

[59] Zhang Y., Xu J., Ruan Y.C., Yu M.K., O’Laughlin M., Wise H., Chen D., Tian L., Shi D., Wang J. (2016). Implant-derived magnesium induces local neuronal production of CGRP to improve bone-fracture healing in rats. Nat. Med..

[60] Hamushan M., Cai W., Zhang Y., Lou T., Zhang S., Zhang X., Cheng P., Zhao C., Han P. (2020). High-purity magnesium pin enhances bone consolidation in distraction osteogenesis model through activation of the VHL/HIF-1α/VEGF signaling. J. Biomater. Appl..

[61] Gu X.-N., Li S.-S., Li X.-M., Fan Y.-B. (2014). Magnesium based degradable biomaterials: A review. Front. Mater. Sci..

[62] Ma J., Thompson M., Zhao N., Zhu D. (2014). Similarities and differences in coatings for magnesium-based stents and orthopaedic implants. J. Orthop. Translat.

[63] Jo J.H., Hong J.Y., Shin K.S., Kim H.E., Koh Y.H. (2012). Enhancing biocompatibility and corrosion resistance of Mg implants via surface treatments. J. Biomater. Appl..

[64] Cheng S., Wang W., Wang D., Li B., Zhou J., Zhang D., Liu L., Peng F., Liu X., Zhang Y. (2020). An in vitro and in vivo comparison of Mg(OH)2-, MgF2- and HA-coated Mg in degradation and osteointegration. Biomater. Sci..

[65] Cheng S., Zhang D., Li M., Liu X., Zhang Y., Qian S., Peng F. (2021). Osteogenesis, angiogenesis and immune response of Mg-Al layered double hydroxide coating on pure Mg. Bioact. Mater..

[66] Liang D.Y., Liang P.C., Yi Q.Q., Sha S., Shi J.F., Chang Q. (2021). Copper coating formed by micro-arc oxidation on pure Mg improved antibacterial activity, osteogenesis, and angiogenesis in vivo and in vitro. Biomed. Microdevices.

[67] Witte F., Kaese V., Haferkamp H., Switzer E., Meyer-Lindenberg A., Wirth C.J., Windhagen H. (2005). In vivo corrosion of four magnesium alloys and the associated bone response. Biomaterials.

[68] Salahshoor M., Guo Y. (2012). Biodegradable Orthopedic Magnesium-Calcium (MgCa) Alloys, Processing, and Corrosion Performance. Materials.

[69] Chen Q., Thouas G.A. (2015). Metallic implant biomaterials. Mater. Sci. Eng. R Rep..

[70] Fattah-alhosseini A., Molaei M., Nouri M., Babaei K. (2022). Antibacterial activity of bioceramic coatings on Mg and its alloys created by plasma electrolytic oxidation (PEO): A review. J. Magnes. Alloys.

[71] Štrbák M., Kajánek D., Knap V., Florková Z., Pastorková J., Hadzima B., Goraus M. (2022). Effect of Plasma Electrolytic Oxidation on the Short-Term Corrosion Behaviour of AZ91 Magnesium Alloy in Aggressive Chloride Environment. Coatings.

[72] Ralston K.D., Birbilis N. (2010). Effect of Grain Size on Corrosion: A Review. Corrosion.

[73] Han H.S., Jun I., Seok H.K., Lee K.S., Lee K., Witte F., Mantovani D., Kim Y.C., Glyn-Jones S., Edwards J.R. (2020). Biodegradable Magnesium Alloys Promote Angio-Osteogenesis to Enhance Bone Repair. Adv. Sci..

[74] Li D., Yuan Q., Yu K., Xiao T., Liu L., Dai Y., Xiong L., Zhang B., Li A. (2019). Mg-Zn-Mn alloy extract induces the angiogenesis of human umbilical vein endothelial cells via FGF/FGFR signaling pathway. Biochem. Biophys. Res. Commun..

[75] Zhang D., Ni N., Su Y., Miao H., Tang Z., Ji Y., Wang Y., Gao H., Ju Y., Sun N. (2020). Targeting Local Osteogenic and Ancillary Cells by Mechanobiologically Optimized Magnesium Scaffolds for Orbital Bone Reconstruction in Canines. ACS Appl. Mater. Interfaces.

[76] Zhang X., Huang P., Jiang G., Zhang M., Yu F., Dong X., Wang L., Chen Y., Zhang W., Qi Y. (2021). A novel magnesium ion-incorporating dual-crosslinked hydrogel to improve bone scaffold-mediated osteogenesis and angiogenesis. Mater. Sci. Eng. C Mater. Biol. Appl..

[77] Sun T.W., Yu W.L., Zhu Y.J., Yang R.L., Shen Y.Q., Chen D.Y., He Y.H., Chen F. (2017). Hydroxyapatite Nanowire@Magnesium Silicate Core-Shell Hierarchical Nanocomposite: Synthesis and Application in Bone Regeneration. ACS Appl. Mater. Interfaces.

[78] Shah F.A., Trobos M., Thomsen P., Palmquist A. (2016). Commercially pure titanium (cp-Ti) versus titanium alloy (Ti6Al4V) materials as bone anchored implants—Is one truly better than the other?. Mater. Sci. Eng. C Mater. Biol. Appl..

[79] Bosshardt D.D., Chappuis V., Buser D. (2017). Osseointegration of titanium, titanium alloy and zirconia dental implants: Current knowledge and open questions. Periodontol. 2000.

[80] Gao P., Fan B., Yu X., Liu W., Wu J., Shi L., Yang D., Tan L., Wan P., Hao Y. (2020). Biofunctional magnesium coated Ti6Al4V scaffold enhances osteogenesis and angiogenesis in vitro and in vivo for orthopedic application. Bioact. Mater..

[81] Li X., Gao P., Wan P., Pei Y., Shi L., Fan B., Shen C., Xiao X., Yang K., Guo Z. (2017). Novel Bio-Functional Magnesium Coating on Porous Ti6Al4V Orthopaedic Implants: In vitro and In vivo Study. Sci. Rep..

[82] Ferraris S., Spriano S. (2016). Antibacterial titanium surfaces for medical implants. Mater. Sci. Eng. C Mater. Biol. Appl..

[83] Yu Y., Jin G., Xue Y., Wang D., Liu X., Sun J. (2017). Multifunctions of dual Zn/Mg ion co-implanted titanium on osteogenesis, angiogenesis and bacteria inhibition for dental implants. Acta Biomater..

[84] Ma L., Cheng S., Ji X., Zhou Y., Zhang Y., Li Q., Tan C., Peng F., Zhang Y., Huang W. (2020). Immobilizing magnesium ions on 3D printed porous tantalum scaffolds with polydopamine for improved vascularization and osteogenesis. Mater. Sci. Eng. C Mater. Biol. Appl..

[85] Chen C., Hao Y., Bai X., Ni J., Chung S.-M., Liu F., Lee I.-S. (2019). 3D printed porous Ti6Al4V cage: Effects of additive angle on surface properties and biocompatibility; bone ingrowth in Beagle tibia model. Mater. Des..

[86] Wang H., Su K., Su L., Liang P., Ji P., Wang C. (2019). Comparison of 3D-printed porous tantalum and titanium scaffolds on osteointegration and osteogenesis. Mater. Sci. Eng. C.

[87] Jackson S.F., Randall J.T. (1956). The fine structure of bone. Nature.

[88] Kazakova G., Safronova T., Golubchikov D., Shevtsova O., Rau J.V. (2021). Resorbable Mg(2+)-Containing Phosphates for Bone Tissue Repair. Materials.

[89] Bohner M., Galea L., Doebelin N. (2012). Calcium phosphate bone graft substitutes: Failures and hopes. J. Eur. Ceram. Soc..

[90] Deng L., Li D., Yang Z., Xie X., Kang P. (2017). Repair of the calvarial defect in goat model using magnesium-doped porous hydroxyapatite combined with recombinant human bone morphogenetic protein-2. Biomed. Mater. Eng..

[91] Canullo L., Heinemann F., Gedrange T., Biffar R., Kunert-Keil C. (2013). Histological evaluation at different times after augmentation of extraction sites grafted with a magnesium-enriched hydroxyapatite: Double-blinded randomized controlled trial. Clin. Oral. Implants Res..

[92] Crespi R., Cappare P., Gherlone E. (2009). Dental implants placed in extraction sites grafted with different bone substitutes: Radiographic evaluation at 24 months. J. Periodontol..

[93] Crespi R., Cappare P., Gherlone E. (2009). Magnesium-enriched hydroxyapatite compared to calcium sulfate in the healing of human extraction sockets: Radiographic and histomorphometric evaluation at 3 months. J. Periodontol..

[94] Crespi R., Mariani E., Benasciutti E., Capparè P., Cenci S., Gherlone E. (2009). Magnesium-Enriched Hydroxyapatite versus Autologous Bone in Maxillary Sinus Grafting: Combining Histomorphometry With Osteoblast Gene Expression Profiles Ex Vivo. J. Periodontol..

[95] Ostrowski N., Roy A., Kumta P.N. (2016). Magnesium Phosphate Cement Systems for Hard Tissue Applications: A Review. ACS Biomater. Sci. Eng..

[96] Tarafder S., Balla V.K., Davies N.M., Bandyopadhyay A., Bose S. (2013). Microwave-sintered 3D printed tricalcium phosphate scaffolds for bone tissue engineering. J. Tissue Eng. Regen. Med..

[97] Wang M., Yu Y., Dai K., Ma Z., Liu Y., Wang J., Liu C. (2016). Improved osteogenesis and angiogenesis of magnesium-doped calcium phosphate cement via macrophage immunomodulation. Biomater. Sci..

[98] Wu X., Dai H., Yu S., Zhao Y., Long Y., Li W., Tu J. (2020). Magnesium Calcium Phosphate Cement Incorporating Citrate for Vascularized Bone Regeneration. ACS Biomater. Sci. Eng..

[99] Liu C., Shao H., Chen F., Zheng H. (2006). Rheological properties of concentrated aqueous injectable calcium phosphate cement slurry. Biomaterials.

[100] Zhang X., Zhu Y., Cao L., Wang X., Zheng A., Chang J., Wu J., Wen J., Jiang X., Li H. (2018). Alginate-aker injectable composite hydrogels promoted irregular bone regeneration through stem cell recruitment and osteogenic differentiation. J. Mater. Chem. B.

[101] Bose S., Tarafder S., Bandyopadhyay A. (2017). Effect of Chemistry on Osteogenesis and Angiogenesis towards Bone Tissue Engineering Using 3D Printed Scaffolds. Ann. Biomed. Eng..

[102] Lu W., Duan W., Guo Y., Ning C. (2012). Mechanical properties and in vitro bioactivity of Ca5(PO4)2SiO4 bioceramic. J. Biomater. Appl..

[103] Wu Q., Xu S., Wang F., He B., Wang X., Sun Y., Ning C., Dai K. (2021). Double-edged effects caused by magnesium ions and alkaline environment regulate bioactivities of magnesium-incorporated silicocarnotite in vitro. Regen. Biomater..

[104] Mousa M., Evans N.D., Oreffo R.O.C., Dawson J.I. (2018). Clay nanoparticles for regenerative medicine and biomaterial design: A review of clay bioactivity. Biomaterials.

[105] Jin S., Xia X., Huang J., Yuan C., Zuo Y., Li Y., Li J. (2021). Recent advances in PLGA-based biomaterials for bone tissue regeneration. Acta Biomater..

[106] Araujo-Pires A.C., Mendes V.C., Ferreira-Junior O., Carvalho P.S., Guan L., Davies J.E. (2016). Investigation of a Novel PLGA/CaP Scaffold in the Healing of Tooth Extraction Sockets to Alveolar Bone Preservation in Humans. Clin. Implant. Dent. Relat. Res..

[107] Kim D.S., Lee J.K., Jung J.W., Baek S.W., Kim J.H., Heo Y., Kim T.H., Han D.K. (2021). Promotion of Bone Regeneration Using Bioinspired PLGA/MH/ECM Scaffold Combined with Bioactive PDRN. Materials.

[108] Liu W., Guo S., Tang Z., Wei X., Gao P., Wang N., Li X., Guo Z. (2020). Magnesium promotes bone formation and angiogenesis by enhancing MC3T3-E1 secretion of PDGF-BB. Biochem. Biophys. Res. Commun..

[109] Wei X., Zhou W., Tang Z., Wu H., Liu Y., Dong H., Wang N., Huang H., Bao S., Shi L. (2023). Magnesium surface-activated 3D printed porous PEEK scaffolds for in vivo osseointegration by promoting angiogenesis and osteogenesis. Bioact. Mater..

[110] Zhao Q., Tang H., Ren L., Wei J. (2020). In Vitro Apatite Mineralization, Degradability, Cytocompatibility and In Vivo New Bone Formation and Vascularization of Bioactive Scaffold of Polybutylene Succinate/Magnesium Phosphate/Wheat Protein Ternary Composite. Int. J. Nanomed..

[111] Kopeček J. (2007). Hydrogel biomaterials: A smart future?. Biomaterials.

[112] Shi W., Huang J., Fang R., Liu M. (2020). Imparting Functionality to the Hydrogel by Magnetic-Field-Induced Nano-Assembly and Macro-Response. ACS Appl. Mater. Interfaces.

[113] Lee K.Y., Mooney D.J. (2001). Hydrogels for Tissue Engineering. Chem. Rev..

[114] Chen R., Chen H.B., Xue P.P., Yang W.G., Luo L.Z., Tong M.Q., Zhong B., Xu H.L., Zhao Y.Z., Yuan J.D. (2021). HA/MgO nanocrystal-based hybrid hydrogel with high mechanical strength and osteoinductive potential for bone reconstruction in diabetic rats. J. Mater. Chem. B.

[115] Yin J., Yan M., Wang Y., Fu J., Suo H. (2018). 3D Bioprinting of Low-Concentration Cell-Laden Gelatin Methacrylate (GelMA) Bioinks with a Two-Step Cross-Linking Strategy. ACS Appl. Mater. Interfaces.

[116] Ding Z., Han H., Fan Z., Lu H., Sang Y., Yao Y., Cheng Q., Lu Q., Kaplan D.L. (2017). Nanoscale Silk-Hydroxyapatite Hydrogels for Injectable Bone Biomaterials. ACS Appl. Mater. Interfaces.

[117] Luo R., Huang Y., Yuan X., Yuan Z., Zhang L., Han J., Zhao Y., Cai Q. (2021). Controlled co-delivery system of magnesium and lanthanum ions for vascularized bone regeneration. Biomed. Mater..

[118] Jing X., Xu C., Su W., Ding Q., Ye B., Su Y., Yu K., Zeng L., Yang X., Qu Y. (2022). Photosensitive and Conductive Hydrogel Induced Innerved Bone Regeneration for Infected Bone Defect Repair. Adv. Healthc. Mater..

[119] Xu Y., Xu C., He L., Zhou J., Chen T., Ouyang L., Guo X., Qu Y., Luo Z., Duan D. (2022). Stratified-structural hydrogel incorporated with magnesium-ion-modified black phosphorus nanosheets for promoting neuro-vascularized bone regeneration. Bioact. Mater..

[120] Vishnu Priya M., Sivshanmugam A., Boccaccini A.R., Goudouri O.M., Sun W., Hwang N., Deepthi S., Nair S.V., Jayakumar R. (2016). Injectable osteogenic and angiogenic nanocomposite hydrogels for irregular bone defects. Biomed. Mater..

[121] Liu C., Yang G., Zhou M., Zhang X., Wu X., Wu P., Gu X., Jiang X. (2021). Magnesium Ammonium Phosphate Composite Cell-Laden Hydrogel Promotes Osteogenesis and Angiogenesis In Vitro. ACS Omega.

[122] Han Y., Li Y., Zeng Q., Li H., Peng J., Xu Y., Chang J. (2017). Injectable bioactive akermanite/alginate composite hydrogels for in situ skin tissue engineering. J. Mater. Chem. B.

[123] Tang Y., Lin S., Yin S., Jiang F., Zhou M., Yang G., Sun N., Zhang W., Jiang X. (2020). In situ gas foaming based on magnesium particle degradation: A novel approach to fabricate injectable macroporous hydrogels. Biomaterials.

[124] Wang Z., Hui A., Zhao H., Ye X., Zhang C., Wang A., Zhang C. (2020). A Novel 3D-bioprinted Porous Nano Attapulgite Scaffolds with Good Performance for Bone Regeneration. Int. J. Nanomed..

[125] Chaya A., Yoshizawa S., Verdelis K., Myers N., Costello B.J., Chou D.T., Pal S., Maiti S., Kumta P.N., Sfeir C. (2015). In vivo study of magnesium plate and screw degradation and bone fracture healing. Acta Biomater..

[126] Globig P., Willumeit-Römer R., Martini F., Mazzoni E., Luthringer-Feyerabend B.J.C. (2022). Slow degrading Mg-based materials induce tumor cell dormancy on an osteosarcoma-fibroblast coculture model. Bioact. Mater..

[127] Zhang X., Zu H., Zhao D., Yang K., Tian S., Yu X., Lu F., Liu B., Yu X., Wang B. (2017). Ion channel functional protein kinase TRPM7 regulates Mg ions to promote the osteoinduction of human osteoblast via PI3K pathway: In vitro simulation of the bone-repairing effect of Mg-based alloy implant. Acta Biomater..

[128] Lin S., Yin S., Shi J., Yang G., Weng X., Zhang W., Zhou M., Jiang X. (2022). Orchestration of energy metabolism and osteogenesis by Mg^2+^ facilitates low-dose BMP-2-driven regeneration. Bioact. Mater..

[129] Wang Y., Kankala R.K., Ou C., Chen A., Yang Z. (2022). Advances in hydrogel-based vascularized tissues for tissue repair and drug screening. Bioact. Mater..

[130] Song W., Fhu C.W., Ang K.H., Liu C.H., Johari N.A., Lio D., Abraham S., Hong W., Moss S.E., Greenwood J. (2015). The fetal mouse metatarsal bone explant as a model of angiogenesis. Nat. Protoc..

[131] Suri C., Jones P.F., Patan S., Bartunkova S., Maisonpierre P.C., Davis S., Sato T.N., Yancopoulos G.D. (1996). Requisite role of angiopoietin-1, a ligand for the TIE2 receptor, during embryonic angiogenesis. Cell.

